# Overall compilation of adverse effects of non-steroidal anti-inflammatory drugs: a hypothesis-free systematic investigation using a nationwide cohort study

**DOI:** 10.3389/fphar.2025.1539328

**Published:** 2025-04-02

**Authors:** Hyein Han, Du Hyun Ro, Hyuk-Soo Han, Sungho Won

**Affiliations:** ^1^ Department of Public Health Sciences, Seoul National University, Seoul, Republic of Korea; ^2^ Department of Orthopaedic Surgery, Seoul National University Hospital, Seoul, Republic of Korea; ^3^ CONNECTEVE Co., Ltd, Seoul, Republic of Korea; ^4^ Innovative Medical Technology Research Institute, Seoul National University Hospital, Seoul, Republic of Korea; ^5^ Department of Orthopaedic Surgery, Seoul National University College of Medicine, Seoul, Republic of Korea; ^6^ Interdisciplinary Program of Bioinformatics, Seoul National University, Seoul, Republic of Korea; ^7^ Institute of Health and Environment, Seoul National University, Seoul, Republic of Korea; ^8^ RexSoft Inc., Seoul, Republic of Korea

**Keywords:** hypothesis-free investigation, non-steroidal anti-inflammatory drugs, adverse effects, time-extended survival analysis, self-controlled case-series studies

## Abstract

**Background:**

Non-steroidal anti-inflammatory drugs (NSAIDs) are widely used for osteoarthritis (OA), despite various adverse effects (AEs). Previous studies were often limited by small sample sizes, a focus on only predefined outcomes, and an imbalanced research coverage across NSAID subtypes. These factors can cause confirmation or heterogeneity bias, and in clinical practice, focusing on only well-known AEs may lead to the overlooking of other potential AEs. To address this, we conducted a hypothesis-free screening of AEs within a large, single cohort.

**Methods:**

Using a nationwide South Korean cohort, we selected 888,909 newly diagnosed OA patients with health screening data between 2010 and 2014. The first three characters of ICD codes were considered as potential AEs and their effects were evaluated. To reduce reverse-causation bias, we first used chi-square and Poisson tests to identify significant indications, and excluded the corresponding ICD codes. Time-dependent survival analysis was conducted, defining NSAID users as patients with any annual medication possession ratio (MPR) ≥ 0.1. Additionally, a self-controlled case series analysis was conducted, defining the risk period as up to 6 months after NSAID intake. Further, we assessed the association between five NSAID subtypes (aceclofenac, meloxicam, loxoprofen, celecoxib, and naproxen) and AEs, and compared their adjusted hazard ratios (aHRs) with each other.

**Results:**

We confirmed previously reported AEs (e.g., anemia, cerebrovascular and cardiorenal diseases). The risk of nephrotoxicity varied significantly by NSAID type, with loxoprofen (aHR = 3.95 [95% CI, 1.56–10.00]), celecoxib (aHR = 2.44 [95% CI, 1.68–3.53]), and naproxen (aHR = 4.7 [95% CI, 2.16–10.24]) showing statistically comparable risks, all of which were significantly higher than that of meloxicam (aHR = 1.22 [95% CI, 0.68–2.19]).

**Conclusion:**

Our findings enhance the understanding of NSAID safety profiles by identifying dose–response and duration–time AEs. They also contribute to better diagnosis and management of AEs while providing valuable guidelines for both patients and clinicians.

## Introduction

Non-steroidal anti-inflammatory drugs (NSAIDs) are widely used to manage and treat various conditions, including acute pain, fever, and chronic inflammation, particularly osteoarthritis (OA) (Wallace, 1997; Zhang et al., 2008). A significant number of older patients depend on long-term NSAID treatment to manage their symptoms. Lee et al. found that 87.7% of patients had been treated with NSAIDs for over 3 months, with approximately half (47.2%) receiving high doses (Lee et al., 2016). Since patients with OA are typically older adults with two to three comorbidities (Rahman et al., 2013; Sharma et al., 2016), careful consideration is necessary when prescribing NSAIDs.

Furthermore, the diversity of patient comorbidities and unshared medical histories between hospitals complicates the attribution of adverse events (AEs) to NSAIDs, leading to an underestimation of their risks. An inaccurate diagnosis of AEs can also cause unnecessary polypharmacy or drug interactions. Therefore, a comprehensive understanding of AEs is essential for clinicians and patients.

NSAIDs exhibit variability in their specificity for cyclooxygenase (COX) enzymes, particularly COX-1 and COX-2, which influence the types of AEs that can occur (Dubois et al., 1998; Smith et al., 2000; Mukherjee et al., 2001; Grosser et al., 2006). COX-2 inhibitors theoretically increase cardiovascular (CV) risk by decreasing the production of vasodilatory and anti-aggregatory prostacyclin, potentially increasing prothrombotic activity (Mukherjee et al., 2001; Grosser et al., 2006). Conversely, COX-1 is implicated in platelet aggregation, gastric mucosal integrity, and kidney function (Scheiman, 1996; Dubois et al., 1998; Smith et al., 2000).

Extensive research on NSAIDs over a prolonged period has revealed the occurrence of multiple AEs (Supplementary Table S1). Specifically, the higher risk of gastric or renal toxicity with non-selective NSAIDs aligns with their pharmacological mechanisms (Deeks et al., 2002; Castellsague et al., 2012; Bhala et al., 2013; Jarupongprapa et al., 2013; Ungprasert et al., 2015; Wang and Li, 2022). However, conflicting results regarding the same AEs, such as CV effects, have been reported. The withdrawal of rofecoxib (an FDA-approved COX-2 inhibitor) owing to serious CV risks led to investigations of other COX-2 inhibitors (Dieppe et al., 2004; Arellano, 2005); however, variable results in different studies often exacerbated confusion rather than providing clarity (Caldwell et al., 2006; Kearney et al., 2006; McGettigan and Henry, 2011; Trelle et al., 2011; Bhala et al., 2013; Essex et al., 2013; Varas-Lorenzo et al., 2013; De Vecchis et al., 2014; Bally et al., 2017; Gunter et al., 2017; Martín Arias et al., 2019; Cheng et al., 2021). Consequently, randomized controlled trials (RCTs) confirmed that celecoxib, another COX-2 inhibitor, was not more hazardous than non-selective NSAIDs (Nissen et al., 2016; MacDonald et al., 2017). However, these RCTs had notable limitations, including higher withdrawal rates and a lower daily dose of celecoxib compared with those of other drugs (FitzGerald, 2017; Schjerning et al., 2020). Additionally, relatively small sample sizes hindered the ability to compare AEs among various NSAIDs.

Prior studies often focused on only predefined NSAID-related outcomes, often pooling information on AEs from multiple studies. Moreover, there is significant variation in the number of research on different NSAIDs, with some subtypes having limited studies. These factors can lead to confirmation bias and heterogeneity, affecting the validity of the findings. Furthermore, in clinical settings, the tendency to focus on well-known AEs may cause other potential AEs to be overlooked, which could lead to severe consequences for elderly patients.

The objective of this study was to overcome these limitations by examining a broad range of AEs including various subtypes in a single large study population devoid of preconceived assumptions. We aimed to provide deeper insights into AE risks and contribute to more refined safety guidelines for clinical practice.

## Methods

### Data source

In this study, we utilized the claims dataset obtained from the National Health Insurance Service (NHIS) in South Korea, which ensures comprehensive healthcare coverage and collects health records for over 99.6% of the Korean population (Ro et al., 2019). These anonymized records, available for research, include demographic details, diagnoses, prescriptions, and health screening data of individuals.

### Study cohorts

For discovery and sensitivity analyses, we examined two specific cohorts: the customized cohort (CC) and the national sample cohort (NSC), both sourced from the NHIS. The CC, derived from the pre-established NHIS data based on researcher-specified criteria (Kyoung and Kim, 2022), provides unrestricted access to clinical and demographic data but limits prescription information to only the drugs requested. Due to its larger and more representative sample size, the CC was used for discovery analysis. The NSC, a randomly selected subset representing 2% of the national population (Lee et al., 2017; Kyoung and Kim, 2022), contains comprehensive records for all medications (Lee et al., 2017). We used the NSC for the sensitivity analysis to assess whether the results from the discovery analysis remained consistent when additional adjustments for concomitant medications were made.

### Inclusion criteria

We focused on patients diagnosed with OA, classified under International Classification of Diseases (ICD) codes M15–M19 from 2010 to 2014. The criteria for inclusion were as follows: no prior OA diagnosis from 2002 to 2009, age over 50 years, availability of health screening data without missing values, and no history of rheumatoid arthritis (ICD codes M05, M06), infectious or inflammatory arthritis (ICD codes M00–03, M10-14), traumatic arthritis or prior fractures (ICD codes S82, M17.2, M17.3), or osteonecrosis (ICD code M87).

### Outcomes

We categorized AEs using the first three characters of ICD codes, with each unique three-character ICD code treated as a distinct potential AE related to NSAID use under the following conditions.

1. We excluded codes unrelated to our main exposure, as listed in Supplementary Table S2.
2. Only ICD codes with more than 500 observed events among study participants were included to ensure sufficient statistical power.
3. We removed ICD codes showing evidence of reverse causation, where the indications for NSAID use could generate statistically significant results. Reverse causation was assessed with the two methods.

3-1. For each ICD code, we calculated the prevalence of NSAID prescription on the same date conditional on the diagnoses with the ICD, and it was compared with the marginal prevalence of NSAID prescription. If the ICD code is an indication for NSAID use, the former is expected to be larger than the latter. The conditional probability of drug prescription given a specific diagnosis code is calculated as follows:

$$P\left(\text{NSAID prescription}|\text{ICD diagnosis}\right)=\frac{N\text{umber}\;\text{of}\;\text{NSAID}\;\text{prescriptions}\;\text{issued}\;\text{on}\;\text{the}\;\text{day}\;the\;\text{ICD}\;\text{code}\;\text{was}\;\text{recorded}}{N\text{umber}\;\text{of}\;times\;the\;\text{ICD}\;\text{codes}\;\text{were}\;\text{recorded}}$$

The latter during the study period was 16.16%, and it was calculated as

$$P\left(\text{NSAID prescription}\right)=\frac{N\text{umber}\;\text{of}\;\text{NSAID}\;\text{prescriptions}\;\text{occurring}\;\text{on}\;\text{the}\;\text{same}\;\text{day}}{N\text{umber}\;\text{of}\;\text{overall}\;\text{hospital}\;\text{visits}}$$

Chi-square test was used to compare it with $P\left(\text{NSAID prescription}|\text{ICD diagnosis}\right)$ at the 0.05 significance level. Consequently, prescription rates for 83 ICD codes exceeded the average (Supplementary Table S3).

3-2. If certain ICD codes are indications for NSAID use, it is expected that the number of prescriptions increased significantly after a recorded diagnosis. For each ICD, we compared its prevalence 10 days before an NSAID prescription with its prevalence 10 days after NSAID prescription by Poisson test. This comparison led to the identification of 183 codes (Supplementary Table S4), which were subsequently removed from the analyses. After these filters, 246 three-digit ICD codes remained and were used as potential AEs for our statistical analyses.

For each potential AE, its occurrence was detected using the corresponding ICD codes. We considered events occurring 1 year after the index date to prevent immortal time bias. In Korea, NSAIDs are usually co-prescribed with gastroprotective medicine to prevent gastrointestinal (GI) complications (Jeong et al., 2023). These prescriptions should be accompanied by GI-related ICD codes such as K20–K31, and GI events occurring on the same day as an NSAID prescription were not considered AEs. After these filters, 246 three-digit ICD codes remained and were used as potential AEs for our statistical analyses.

### Study design and main exposure

We used multiple analyses to screen for AEs. For discovery purposes, we used CC data from 888,909 newly diagnosed (2010-2014) OA patients, employing both cohort and case-only study designs. The main exposure was NSAID prescriptions associated with OA, considering various subtypes classified according to the anatomical therapeutic chemical (ATC) classification system (detailed in Supplementary Table S5). The observation period ended in 2018.

In the cohort design, each participant prescribed NSAIDs was matched with five non-NSAID users based on propensity score matching with age, sex, and body mass index (BMI). The index date was set at the issuance of the first NSAID prescription following an OA diagnosis; for matched non-users, this date aligned with their medicated counterparts. Collectively, we included 120,691 NSAID users and 548,978 non-users. Exposed groups were defined using medication possession ratios (MPRs), which are used to quantify the proportion of days a medication was prescribed annually, serving as a measure of drug compliance. Patients were categorized into non-medication groups (MPR <0.1 throughout the observation period) and medication groups (MPR ≥0.1 at any point during the study).

In the case-only design, all participants (N = 888,909) were included without segregation into medication/control groups, with the observation start 1 year before the OA diagnosis to avoid potential early clustering of AEs.

For sensitivity analyses, significant results from the discovery dataset (CC) were replicated after adjusting concurrent treatments using a separate NSC dataset. We expanded the study to include patients newly diagnosed with OA from 2010 to 2018 to ensure a large sample size, using the same criteria and index dates as the original dataset, ultimately involving 23,138 medicated and 60,669 non-medicated patients.

### Covariates

To adjust for confounding effects in cohort design, several potential confounders such as age, sex, BMI, Charlson comorbidity index (CCI), income, and residence were incorporated as covariates. Specifically, age, BMI, and CCI were included as continuous-scale covariates, whereas sex, income, and residence were considered as categorical variables. Income was divided into 20 groups, and residence was classified as a metropolitan city, small city, or rural area. These variables were adjusted for at the ordinal and nominal scales, respectively.

Case-only design inherently accounts for covariates within individuals and, thus, does not require additional adjustments. The OA diagnosis date was used to account for changes in the time-varying condition related to OA itself.

In the sensitivity analyses, we used the same design and covariates as the cohort study, with the additional adjustment for concomitant drugs related to the classified results.

### Statistical analyses

For demographic statistics, continuous variables were tested using t-tests, and categorical variables were tested using Chi-square tests.

For the cohort study, we conducted Cox proportional hazard analyses with the onset age of potential AEs as the outcome. For each potential AE, a 1-year washout period was applied, and participants with any event during that period were further filtered out on a per-AE basis. Annual MPRs were treated as time-dependent exposures, leading to the execution of an extended Cox proportional hazards model. The effect of NSAID on each AE was estimated after adjusting for covariates. Subsequently, we identified an association between each identified AE and the five most commonly prescribed NSAIDs: aceclofenac, meloxicam, loxoprofen, celecoxib, and naproxen. We used the same Cox proportional hazard model, with the primary exposure variable shifted to these five types of NSAIDs. Subsequently, pairwise comparison was performed using a one-sided Wald test.

For the case-only study, self-controlled case series (SCCS) analyses were performed to explore the significant association of each potential AE (Ahn et al., 2023). Similar to the cohort study, a 1-year washout period was implemented. In the SCCS framework, the risk period was defined from the initiation of NSAID use, extending to 6 months after cessation of the medication. Within this timeframe, “current use” was defined as the actual duration of medication use, with an additional 14-day period designated as “recent use.” The remaining days within the risk period were classified as “past use.” To address potential biases arising from reduced observed periods, particularly those resulting from high fatality rates of severe AEs, an extended SCCS model was employed in relevant cases. The ICD codes consistently identified in both analyses were defined as AEs of NSAIDs.

A Bonferroni-adjusted significance level of 0.05 was established. Given the large sample size, we also set an additional threshold of |beta| > 0.2 to ensure substantial differences. These criteria were consistently applied to both cohort and case-only studies, and only results meeting them were considered significant.

**FIGURE 1:**
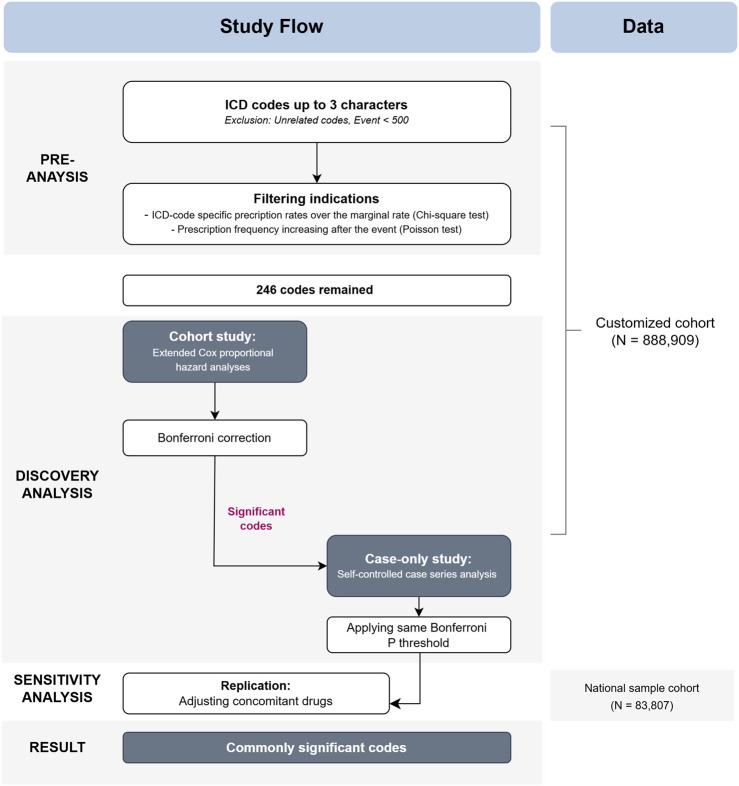
Flow chart of the study design. Abbreviations: ICD, International Statistical Classification of Diseases, 10th Revision.

In the sensitivity analyses, we first conducted a replication using the same Cox proportional model and covariates as in the cohort study from the discovery analysis. We Subsequently, we added drug-related covariates to check if the results remained consistent. Due to the insufficient sample size to perform a case-only design, sensitivity analyses were focused on the cohort study. For clarity, all references to “cohort study” in the following text will specifically refer to the cohort study in the discovery analysis, while “sensitivity analysis” will be used as described. A detailed study flow and diagram are presented in Figure 1 and Supplementary Figure S1.

The statistical significance level was set to 0.05. All analyses were performed using R software (version 4.0; R Project, Vienna, Austria), SAS Enterprise Guide (version 7.15; SAS Institute Inc., Cary, NC, United States), and Rex (version 3.5.3; RexSoft, Seoul, Republic of Korea).

## Results

### Patient characteristics

The study population characteristics are summarized in Table 1. The mean age of the medicated groups was significantly higher than that of the non-medicated groups (P < 0.001). Despite significant differences across all variables, standardized mean differences for matching variables (age, sex, and BMI) were confirmed to be <0.2, ensuring a balance between the groups. Additionally, the frequencies of subtypes and daily doses of prescribed NSAIDs are detailed in Supplementary Figure S2. Aceclofenac was the most prescribed NSAID for OA, followed by meloxicam, loxoprofen, celecoxib, and naproxen.

**TABLE 1:** Descriptive statistics.

	Standardized mean difference	Non-medicated	Medicated	
		(N = 548,978)	(N = 120,691)	P^a^
Age (years)	0.12	57.0 ± 8.6	58.2 ± 8.9	<0.001
Sex	0.06			<0.001
Male		283,788 (51.8%)	58,820 (48.7%)	
Female		264,590 (48.2%)	61,871 (51.3%)	
Body mass index	0.03	24.2 ± 3.1	24.3 ± 3.2	<0.001
Residence				<0.001
Metropolitan city		269,622 (49.2%)	62,986 (52.3%)	
Small city		234,721 (42.9%)	48,336 (40.1%)	
Rural		43,189 (7.9%)	9,181 (7.6%)	
Income		11.7 ± 6.1	11.3 ± 6.1	<0.001
Charlson comorbidity index		1.25 ± 1.7	1.5 ± 1.7	<0.001

Notes: ^a^ P-values were generated by t-test for continuous variables and chi-square test for categorical variables.

### Adverse effects associated with general NSAID treatment

**FIGURE 2:**
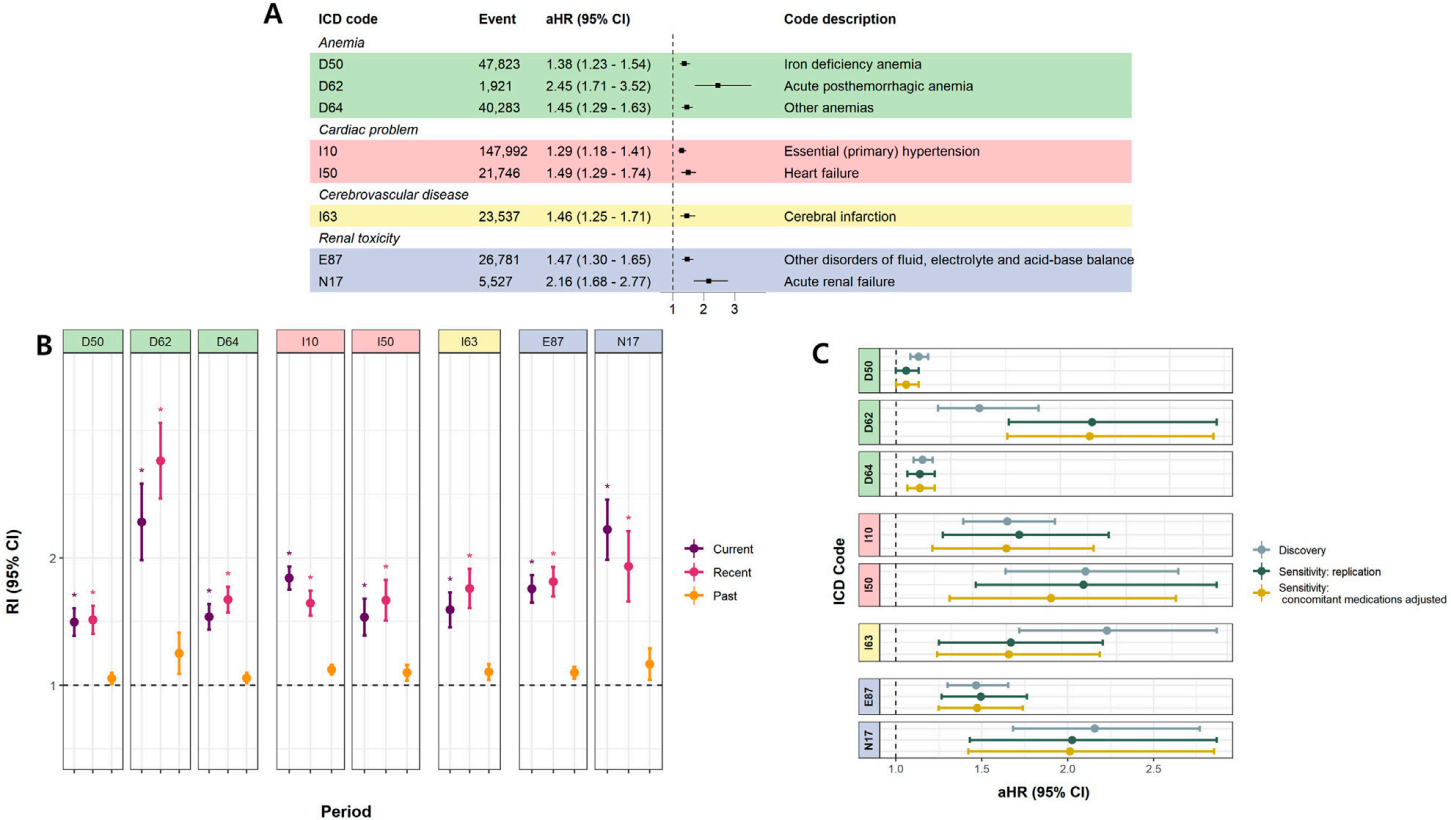
Significant adverse effects of overall NSAIDs **(A)** Cohort study (Time-dependent survival analyses) **(B)** Case only study (self-controlled case series study) **(C)** Sensitivity analyses. **(A)** illustrates the aHRs and 95% CIs for MPR in the time-dependent survival analysis. **(B)** represents RIs and 95% CIs for each period from the SCCS analysis. * denotes significant results (Bonferroni-adjusted P-value <0.05 and |beta| > 0.2). **(A)** and **(B)** were conducted using CC data, and only results significant in both analyses are presented. **(C)** presents the results of the sensitivity analysis. ‘Discovery’ corresponds to the original results from the CC, identical to those in **(A)**. ‘Sensitivity: replication’ shows the aHRs and 95% CIs for MPR when the same covariates (age, sex, BMI, Charlson comorbidity index, income, and residence) are applied to the NSC data. ‘Sensitivity: concomitant medication adjusted’ represents results obtained further adjusting for concomitant medications (see Supplementary Table S6) using NSC data. Abbreviations: aHR, adjusted Hazard ratio; CI, Confindence interval; MPR, Medication possession ratio; SCCS, Self-controlled case series; RI, Relative incidence; CC, Customized cohort; NSC, National sample cohort.

We considered 246 potential outcomes in discovery analyses, with cohort study results for all included ICD codes presented in Supplementary Figure S3. Eight codes demonstrated significance at the 0.0002 level (equivalent to the 0.05 BF-adjusted significance level), consistently showing the same direction of effect. Detailed information on significant AEs is provided in Figure 2. These AEs were categorized based on similarities in diagnosis: anemia (D50, D62, and D64), cardiac effects (I10 and I50), cerebrovascular disease (I63), and renal toxicity (E87 and N17).

The highest adjusted HR (aHR) derived from the cohort study for the general NSAID MPR was observed for acute posthemorrhagic anemia (D62; adjusted HR [aHR] = 2.45, 95% confidence interval [CI] = 1.71–3.52) and acute renal failure (ARF) (N17; aHR = 2.16, 95% CI = 1.68–2.77) (Figure 2A). Consistent with the cohort study, acute posthemorrhagic anemia and ARF exhibited the highest relative incidences (RIs) in the case-only study. The RIs for these diagnoses were significantly higher than one during both current and recent periods, indicating that the incidence of AEs lasted up to 2 weeks post-medication. Elevated RIs became not significant for the past period, from 2 weeks to 6 months post-NSAID intake, for all AEs. (Figure 2B). Additionally, the sensitivity analysis adjusting for concomitant medication did not alter the significance of the results (Figure 2C). The list of concomitant drugs is presented in Supplementary Table S6.

### Adverse effects of individual NSAIDs

**FIGURE 3:**
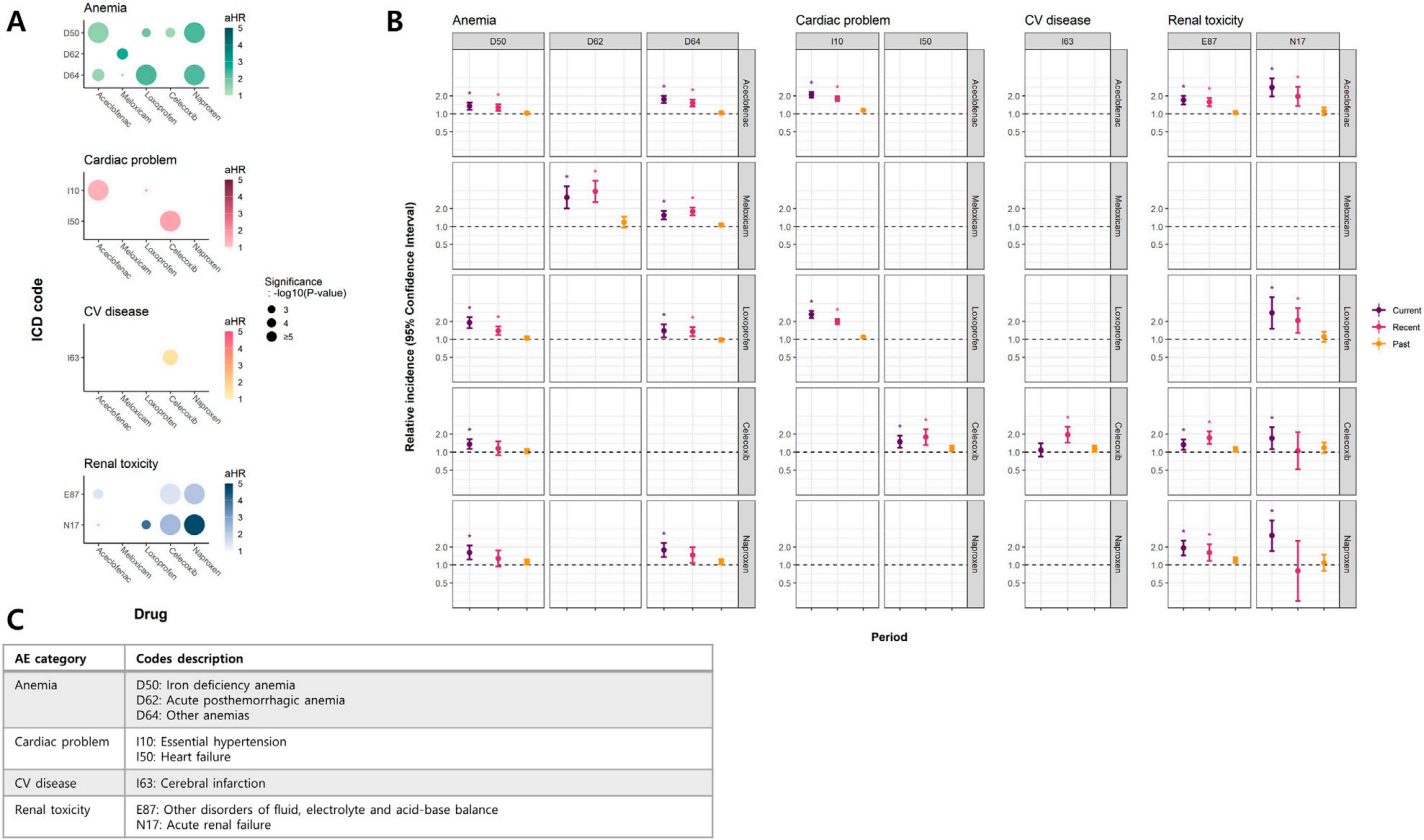
Significant findings in NSAID subtype analyses **(A)** Bubble plot of hazard ratios (HR) in cohort study (survival analyses) **(B)** Case-only study (self-controlled case series study) **(C)** Description of ICD codes with significant results. **(A)** presents a bubble plot of aHRs for MPR obtained from the time-dependent survival analysis. The color intensity of each bubble corresponds to the magnitude of aHR, with darker colors indicating larger aHRs. The size of each bubble reflects the level of significance, with larger bubbles indicating higher certainty. **(B)** shows RIs and 95% CIs for each period from the SCCS analysis. * denotes significant results. Both **(A)** and **(B)** show results for NSAID subtypes that met the significance threshold in both analyses. **(C)** provides ICD codes and their descriptions for the outcomes in **(A)** and **(B)**. Abbreviations: aHR, adjusted Hazard ratio; CI, Confindence interval; MPR, Medication possession ratio; SCCS, Self-controlled case series; RI, Relative incidence; CC, Customized cohort; ICD, International Statistical Classification of Diseases, 10th Revision.

Figure 3 displays the estimated effects of individual NSAIDs, and Supplementary Figure S4 presents the results of the pairwise comparisons. Figure 3A shows the outcomes from the cohort analyses. For anemias, non-selective NSAIDs exhibited significantly higher risks than celecoxib (pairwise P < 0.05). When examining hypertension (I10), the risks associated with all NSAID subtypes were comparable (pairwise P > 0.05), with loxoprofen demonstrating a relatively lower hazard for heart failure (HF) (I50; pairwise P < 0.05). Celecoxib was the only NSAID that showed a significant association with cerebrovascular disease, although the risk was not significantly higher than that of other NSAIDs (pairwise P > 0.05). In terms of renal toxicity, most NSAIDs demonstrated significance, except for meloxicam. Notably, naproxen exhibited a substantially high HR (aHR 4.7, 95% CI 2.16–10.24) (P < 0.0001) (Figure 3A; Supplementary Figure S5); however, the risk was comparable with those of celecoxib and loxoprofen (pairwise P > 0.05). The results from the case-only studies were consistent with those from the cohort ones (Figure 3B).

## Discussion

In this study, we determined which individual NSAIDs were more likely to cause certain AEs and provided evidence that this drug class could be a contributing factor to existing diseases. This study represents the first comprehensive evaluation of AEs across all available NSAID subtypes using a single cohort of large populations and various methods without predefined hypotheses. This approach allows for a multidimensional comparison of both the overall and the individual risks associated with each NSAID-related AE. We focused on AEs associated with both general and specific NSAIDs in OA patients.

To minimize bias, we employed various approaches. First, we identified data-driven indications and excluded them from potential AEs prior to screening. We also recognized that certain variables could act as confounder, necessitating additional adjustments for related outcomes. Therefore, we considered lifestyle factors such as smoking and alcohol consumption as covariates, given their potential role as confounders. The findings remained consistent after these adjustments, with overlapping CIs for aHRs (Supplementary Figure S5). Further, sensitivity analyses accounting for concomitant drugs were also conducted to verify the robustness of our results.

Systematic approaches allowed us to better understand dose–response and duration–time effects of NSAID use. The dose–response effect was used to explore the probability of AEs with prolonged drug intake, while the duration–time effect was used to examine the evolution of AEs following drug administration.

In our subtype analysis, we specifically investigated celecoxib, a selective COX-2 inhibitor, alongside four non-selective NSAIDs: aceclofenac, meloxicam, loxoprofen, and naproxen, which inhibit both COX-1 and COX-2 enzymes but vary in their COX-2 selectivity. Our findings highlighted several clinically important implications. Although the magnitude of the HR was not large, we observed a significant association between various non-selective NSAIDs and anemia-related AEs. These findings corroborate previous studies reporting that non-selective NSAIDs may lead to an increased incidence of anemia (Singh et al., 2006; Essex et al., 2013) or significant decreases in hemoglobin or hematocrit levels (Silverstein et al., 2000). This emphasizes the need for caution with respect to bleeding up to 2 weeks after stopping the medication.

Conversely, stroke- or hemorrhage-related anemia was uniquely associated with celecoxib or meloxicam, respectively, although these did not present a significantly higher risk than other NSAIDs. Unlike several previous studies, our findings did not reveal significant differences in hemorrhage risk regardless of COX selectivity, indicating the need for further research in this area. The CV risk of celecoxib has been a subject of ongoing debate; however, recent studies focusing on low-dose users (200 mg daily) have concluded that the CV risk is comparable to that of other NSAIDs, aligning with our results (Nissen et al., 2016; Chan et al., 2017).

We also observed that the variation in risk was the most pronounced in cardiorenal AEs. Specifically, the overall HR of ARF was among the highest observed (aHR = 2.16, 95% CI = 1.68–2.77), with HR trends varying across each subtype of NSAID. In particular, drug-specific risks were higher and did not differ significantly with COX selectivity. Our HR calculations, based on MPR as a continuous variable, showed that the dose–response effects of celecoxib, naproxen, and loxoprofen were comparable (pairwise P > 0.05). Theoretically, inhibition of COX-1 affects renal hemodynamics, potentially leading to a reduced glomerular filtration rate (GFR) and, consequently, to acute renal injury (Nantel et al., 1999; Moore et al., 2015; Moro et al., 2017). Similarly, inhibition of COX-2 can cause electrolyte imbalance or renal dysfunction (Lucas et al., 2018); however, the risk has not been sufficiently highlighted in clinical practice. While several studies have suggested a lower risk of nephrotoxicity associated with celecoxib compared with that of non-selective NSAIDs (Schneider et al., 2006; Ungprasert et al., 2015; Obeid et al., 2022; Wang and Li, 2022), our findings indicate that long-term use of celecoxib is also highly hazardous. Specifically, for ARF, the RI was significantly higher during the current period, indicating a substantially high incidence of AEs during treatment. Thus, ARF requires close monitoring during drug administration.

Moreover, meloxicam, the second most prescribed NSAID in our study population and not available over the counter, demonstrated noteworthy findings. We did not find an association between meloxicam and cardiorenal events and showed it was less nephrotoxic than naproxen, loxoprofen, or celecoxib (all pairwise P < 0.05). These results suggest that meloxicam may be a safer option for patients with compromised cardiorenal health. Given the limited research on meloxicam, further studies are warranted to comprehensively understand its impact and optimize its use in clinical settings.

In summary, our study presents several novelty findings. We identified the potential risk of anemias, less emphasized compared to other AEs (cardiorenal or CV), and found them to be associated of various types of NSAIDs. Additionally, despite widely prescribed, meloxicam and its oxicam subclass remain relatively under-researched. We screened systemically and provided new insights into meloxicam’s safety profile.

Our study has several strengths. First, we used a structured and meticulous approach. Studies involving widely used drugs such as NSAIDs often run the risk of reverse causation bias. To mitigate this risk, we incorporated statistical procedures designed to filter out potential indications. Second, numerous previous studies have focused on examining the effects of celecoxib on various AEs or comparing them with non-selective NSAIDs. Notably, in almost all comparative cases we reviewed, celecoxib was investigated independently, whereas non-selective NSAIDs were often pooled together as a reference group rather than being assessed separately. This approach may inadvertently lead to confirmation bias toward a specific drug. To mitigate this potential bias, we performed our analysis without any pre-established hypotheses, and compared all subtypes separately.

Despite its strengths, our study has several limitations. First, given the broad range of outcomes we sought to screen, managing and controlling for individual confounders proved challenging. Nevertheless, we employed various approaches and performed sensitivity analyses to minimize the potential bias generated by unobserved confounders. Second, gastric ulcer is a well-known AE of NSAIDs. A previous study reported that gastroprotective agents are co-prescibed in nearly 90% of NSAID prescriptions in South Korea, indicating that these agents could act as confounders (Jeong et al., 2023). Given the high co-prescription rate and resulting multicollinearity, we excluded gastrointestinal codes recorded simultaneously with NSAID prescriptions instead of including gastroprotective agents as covariates. However, these factors complicate the accurate assessment of NSAID-related GI AEs, underscoring the need for further studies using alternative datasets. Third, sample sizes varied considerable across NSAID subtypes (Supplementary Figure S2). In particular, the number of naproxen users was the smallest (Supplementary Figure S2), potentially resulting in relatively lower statistical power. Furthermore, the limited sample size prevented us from conducting sensitivity analyses for individual subtypes in NSC data, although replication analyses for overall NSAIDs were validated. Thus, future studies with larger sample sizes may be needed to confirm the validity of our assumptions and findings for specific NSAIDs subtypes.

## Conclusion

In this study, we comprehensively evaluated AEs associated with NSAIDs and their commonly prescribed subtypes. Utilizing a single large-sample dataset and conducting hypothesis-free analyses with multiple approaches, we enhanced the reliability of our findings, reducing several biases. These can contribute to improving the treatment or diagnosis of AEs that have already occurred and help provide guidelines for both patients and clinicians when prescribing NSAIDs.

## Data Availability

The data used in this study were obtained from the NHIS under the license numbers NHIS-2021-1-339 for CC and NHIS-2023-2-086 for NSC, respectively. Researchers can request the dataset by creating an account and completing the registration process. Additional details about data access are available on the NHIS website (https://nhiss.nhis.or.kr).
