# Supplementary material for: Overall compilation of adverse effects of non-steroidal anti-inflammatory drugs: a hypothesis-free systematic investigation using a nationwide cohort study
Source: Front Pharmacol. 2025 Apr 2;16:1539328. doi: 10.3389/fphar.2025.1539328 (PMC12001242; doi:10.3389/fphar.2025.1539328)
Supplement: Supplementary file 1 [file Supplementaryfile1.docx]

Supplementary Material

Overall compilation of adverse effects of non-steroidal anti-inflammatory drugs: a hypothesis-free systematic investigation using a nationwide cohort study

**Hyein Han, MPH^1^; Du Hyun Ro, MD, PhD^2,3,4,5^; Hyuk-Soo Han, MD, PhD^2,5†^; Sungho Won, PhD^1,6,7,8†^**

^1^Department of Public Health Sciences, Seoul National University, Seoul, Republic of Korea.

^2^Department of Orthopaedic Surgery, Seoul National University Hospital, Seoul, Republic of Korea.

^3^CONNECTEVE Co., Ltd, Seoul, Republic of Korea

^4^Innovative Medical Technology Research Institute, Seoul National University Hospital, Seoul, Republic of Korea

^5^Department of Orthopaedic Surgery, Seoul National University College of Medicine, Seoul, Republic of Korea

^6^Interdisciplinary Program of Bioinformatics, Seoul National University, Seoul, Republic of Korea.

^7^Institute of Health and Environment, Seoul National University, Seoul, Republic of Korea.

^8^RexSoft Inc., Seoul, Republic of Korea

*** Correspondence:**Hyuk-Soo Han, Department of Orthopedic Surgery, Seoul National University Hospital, 101 Daehak-ro, Jongno-gu, Seoul, 03080, Republic of Korea. Email: oshawks7@snu.ac.kr, Tel: +82-2-2072-3212, Fax: +82-2-764-2718

Sungho Won, Department of Public Health Science, Seoul National University, 1 Kwanak-ro, Kwanak-gu, Seoul, 08826, Republic of Korea. Email: won1@snu.ac.kr, Tel: +82-2-880-2714, Fax: +82-303-0942-2714

†These authors contributed equally to this work and should be considered as co-corresponding authors.

**Supplementary Table S1. Summary of Reviewed Meta-analyses reporting on Common Adverse effects of NSAIDs**

| **AE type** | **Author (Year)** | **Number of studies** | **AE subtype** | **Non-selective NSAIDs** | | | | **COX-2 inhibitor** | | **Description** |
| --- | --- | --- | --- | --- | --- | --- | --- | --- | --- | --- |
|  |  |  |  | **Diclofenac** | **Meloxicam** | **Ibuprofen** | **Naproxen** | **Celecoxib** | |  |
|  |  |  |  |  |  |  |  | **(Placebo / nsNSAIDs)** | |  |
| GI | Castellsague et al. (2012) | 28 | Upper gastrointestinal complication | O* (▲) | O* (▲) | O* (▲) | O* (▲) | O* (▲) |  | Most NSAIDs were associated with GI AE. Especially, the risk of celecoxib and aceclofenac were the lowest. |
|  | Bhala et al. (2013) | 757 | bleed | O* (▲) |  | O* (▲) | O* (▲) | O* (▲) | O | All NSAIDs were associated with Upper GI complications and bleeding. However, celecoxib's risk was lower than ibuprofen and naproxen |
|  |  |  | Upper GI complication | O* (▲) |  | O* (▲) | O* (▲) | O* (▲) | O* (▼): |  |
|  |  |  |  |  |  |  |  |  | (Only naproxen, |  |
|  |  |  |  |  |  |  |  |  | Ibuprofen) |  |
|  | Deeks et al. (2002) | 9 | Any GI event |  |  |  |  | O* (▲) | O* (▼) | Celecoxib tended to occur GI events but the risk was lower than non-selective NSAIDs. |
|  |  |  | Abdominal pain | |  |  |  | O | O* (▼) |  |
|  |  |  | Diarrhea |  |  |  |  | O | O |  |
|  |  |  | dyspepsia |  |  |  |  | O | O* (▼) |  |
|  |  |  | nausea |  |  |  |  | O | O |  |
|  |  |  | vomiting |  |  |  |  | O | O |  |
|  | Jarupongprapa et al. (2013) | 9 | GI  complications | |  |  |  |  | O* (▼) | The GI risk was lower in celecoxib users compared to non-selective NSAIDS + Proton pump inhibitor |
| CV | Martín Arias et al. (2019) | 87 | Cerebrovascular events | O* (▲) |  | O* (▲) | O* (▲) | O* (▲) |  | The risk of non-selective NSAIDs were all high in every types of CV. However, only the risk of cerebrovascular disease was significant for celecoxib. Overally, celecoxib (high-dose), diclofenac (high &low does) were associated. |
|  |  |  | Cardiac events | O* (▲) |  | O* (▲) | O* (▲) | O |  |  |
|  |  |  | Vascular events | O* (▲) |  | O* (▲) | O* (▲) | O |  |  |
|  | McGettigan et al. (2011) | 51 | Overall CV risk | O* (▲) | O* (▲) | O* (▲) | O* (▲) | O* (▲) |  | Relative risk for naproxen was the lowest, and diclofenac was the highest. Celecoxib and diclofenac showed significance for all doses, and ibuprofen for high dose. However, the dose-response effect was not significant for naproxen. |
|  |  |  |  |  | (No dose information) | (Only high-dose) | (Non-significant dose-response relationship) |  |  |  |
|  | Gunter, B. R., et al. (2016) | 26 | MI |  |  |  | O | O | O | Celecoxib did not show higher cardiovascular risks compared to both placebo and non-selective NSAIDs. |
|  |  |  | Stroke | O |  |  | O | O | O* (▼) |  |
|  |  |  | CV death |  |  |  | O | O | O |  |
|  | Trelle et al. (2011) | 31 | MI | O |  | O | O | O |  | Celecoxib did not show higher cardiovascular risks compared to both placebo and non-selective NSAIDs. However, diclofenac showed significantly higher risk of stroke compared to placebo. |
|  |  |  | Stroke | O* (▲) |  | O* (▲) | O | O | O* (▼): |  |
|  |  |  |  |  |  |  |  |  | (Only diclofenac) |  |
|  |  |  | CV death | O* (▲) |  | O | O | O | O |  |
|  | Caldwell et al (2006) | 6 | MI |  |  |  |  | O* (▲) |  | Among thrombotic AEs, MI was significantly associated with celecoxib. |
|  |  |  | Stroke |  |  |  |  | O |  |  |
|  |  |  | CV death |  |  |  |  | O |  |  |
|  | Bhala et al. (2013) | 757 | Major vascular events | O* (▲) |  | O | O | O* (▲) | O | For non-selective NSAIDs, only high doses were included in the analysis. Except for naproxen, all NSAIDs were associated with cardiovascular risks, excluding the risk on coronary events of ibuprofen. The risk between celecoxib and other NSAIDs was not significantly different. |
|  |  |  | Major coronary events | O* (▲) |  | O* (▲) | O | O* (▲) | O |  |
|  | Essex et al. (2013) | 89 | Embolic and thrombotic events |  |  |  |  | O | O | The risk of thrombotic diseases is not significantly different between celecoxib and non-selective NSAIDs. |
|  |  |  |  |  |  |  |  |  |  |  |
|  | Cheng et al. (2021) | 21 | CV death |  |  |  |  | O | O | Celecoxib did not significantly increase CV risk when compared to placebo or other NSAIDs, regardless of dosage or duration. |
|  |  |  | MI |  |  |  |  | O | O |  |
|  |  |  | Stroke |  |  |  |  | O | O |  |
|  |  |  | Atrial fibrilation | |  |  |  | O | O |  |
|  |  |  | Arrhythmias |  |  |  |  | O | O |  |
|  |  |  | Angina |  |  |  |  | O | O |  |
|  | Kearney et al. (2006) | 138 | MI |  |  |  |  | O |  | Celecoxib was not associated with all thrombotic events. However, the dose-response effect of celecoxib for serious vascular events was significant. |
|  |  |  | Stroke |  |  |  |  | O |  |  |
|  |  |  | Vascular death |  |  |  |  | O |  |  |
|  | Varas‐Lorenzo et al. (2013) | 25 | MI | O* (▲) |  | O | O | O* (▲) |  | Celecoxib and diclofenac showed association with acute MI. |
|  | Bally et al. (2017) | 8 | MI | O* (▲) |  | O* (▲) | O* (▲) | O | O | MI risks were significant in diclofenac, ibuprofen, and naproxen users. However, the risk comparison between celecoxib and non-selective NSAIDs were not significant. |
| Nephro-toxicity | Essex et al. (2013) | 89 | Hypertension |  |  |  |  | O | O* (▼) | Compared to placebo, celecoxib only exhibited a significant increase in the risk of edema. However, when compared to non-selective NSAIDs, the risks of edema and hypertension were notably lower. There was no significant association with renal impairment when compared to either a placebo or non-selective NSAIDs. |
|  |  |  | Edema |  |  |  |  | O* (▲) | O* (▼) |  |
|  |  |  | Renal impairment |  |  |  |  | O | O |  |
|  | Ungprasert et al. (2015) | 5 | Acute kidney injury | O | O | O* (▲) | O* (▲) | O |  | AKI risk was significant in non-selective NSAID users (including naproxen and ibuprofen), but not in celecoxib users. |
|  | Wang et al. (2022) | 20 | Hypertension | O* (▲) |  | O* (▲) | O | O | O* (▼): | Non-selective NSAIDs were all associated with nephrotoxicity, and the cardio-renal risk of celecoxib was lower than others. |
|  |  |  |  |  |  |  |  |  | (Only ibuprofen) |  |
|  |  |  | Nephrotoxicity | O* (▲) |  | O* (▲) | O* (▲) | O* (▲) | O* (▼): |  |
|  |  |  |  |  |  |  |  |  | (Only naproxen, |  |
|  |  |  |  |  |  |  |  |  | Ibuprofen) |  |
|  |  |  | Edema | O* (▼) |  | O | O | O | O* (▼): |  |
|  |  |  |  |  |  |  |  |  | (Only naproxen) |  |

**Notes:** If the results indicated a significantly higher or lower incidence of AEs compared to the control, we filled the boxes with O* (▲) and O* (▼), respectively. In cases where the difference in incidence was not significant, we used only circle without *. Blank box means that the corresponding drugs were not considered in the study. For non-selective NSAIDs, adverse events were compared to placebo or non-users. In the case of celecoxib, if the control group was non-selective NSAID users, we marked the right box, and if it was placebo or non-users, we marked the left box. If the boxes for non-selective NSAIDs were left blank and only the right boxes for celecoxib were filled, it indicates that the comparison was made solely with non-selective NSAIDs, and no comparison with placebo was conducted for any NSAIDs.

**Abbreviations:** NSAID, Nonsteroidal Anti-Inflammatory Drug; nsNSAID, non-selective Nonsteroidal Anti-Inflammatory Drug; AE, Adverse effect; GI, Gastrointestinal; CV, Cardiovascular; MI, Myocardial infarction;

**Supplementary Table S2. Inclusion/Exclusion of Potential Adverse Events**

| **ICD code** | **Code description** | **Cause of exclusion** |
| --- | --- | --- |
| **Excluded** | | |
| A00-B99 | Certain infectious and parasitic diseases | Unrelated (External disease) |
| F00-F99 | Mental and behavioural disorders | Ambiguity due to complexity of the etiology |
| M00-M99 | Diseases of the musculoskeletal system and connective tissue | Direct correlation with the study population |
| O00-O99 | Pregnancy, childbirth and the puerperium | Unrelated disease to our study population |
| P00-P96 | Certain conditions originating in the perinatal period | Unrelated disease to our study population |
| Q00-Q99 | Congenital malformations, deformations and chromosomal abnormalities | Unrelated disease to our study population |
| R00-R99 | Symptoms, signs and abnormal clinical and laboratory findings, not elsewhere classified | Ambiguity due to the unclassification to specific disease |
| S00-T98 | Injury, poisoning and certain other consequences of external causes | Unrelated (External disease) |
| V01-Y98 | External causes of morbidity and mortality | Unrelated (External disease) |
| Z00-Z99 | Factors influencing health status and contact with health services | Unrelated (External disease) |
| U00-U85 | Codes for special purposes | Unrelated to our study population |
| **Included** | | |
| C00-D48 | Neoplasms |  |
| D50-D89 | Diseases of the blood and blood-forming organs and certain disorders involving the immune mechanism | |
| E00-E90 | Endocrine, nutritional and metabolic disease |  |
| G00-G99 | Diseases of the nervous system |  |
| H00-H59 | Diseases of the eye and adnexa |  |
| H60-H95 | Diseases of the ear and mastoid process |  |
| I00-I99 | Diseases of the circulatory system |  |
| J00-J99 | Diseases of the respiratory system |  |
| K20-K93 | Diseases of the digestive system |  |
| L00-L99 | Diseases of the skin and subcutaneous tissue |  |
| N00-N99 | Diseases of the genitourinary system |  |

**Abbreviations:** ICD, International Statistical Classification of Diseases, 10th Revision;

**Supplementary Table S3. Filtered indications by the prescription rates**

| **ICD code** | **Conditional prescription rate (%) ^a^** | **P ^b^** | **Code description** |
| --- | --- | --- | --- |
| D10 | 17.96 | <.0001 | Benign neoplasm of mouth and pharynx |
| D17 | 16.49 | <.0001 | Benign lipomatous neoplasm |
| D21 | 17.67 | <.0001 | Other benign neoplasms of connective and other soft tissue |
| G24 | 24.51 | <.0001 | Dystonia |
| G43 | 21.27 | <.0001 | Migraine |
| G44 | 21.74 | <.0001 | Other headache syndromes |
| G50 | 21.69 | <.0001 | Disorders of trigeminal nerve |
| G53 | 27 | <.0001 | Cranial nerve disorders in diseases classified elsewhere |
| G54 | 29.61 | <.0001 | Nerve root and plexus disorders |
| G55 | 28.44 | <.0001 | Nerve root and plexus compressions in diseases classified elsewhere |
| G56 | 32.45 | <.0001 | Mononeuropathies of upper limb |
| G57 | 29.05 | <.0001 | Mononeuropathies of lower limb |
| G58 | 33.39 | <.0001 | Other mononeuropathies |
| G64 | 24.83 | <.0001 | Other disorders of peripheral nervous system |
| G90 | 16.47 | 0.0019 | Disorders of autonomic nervous system |
| G95 | 20.2 | <.0001 | Other diseases of spinal cord |
| H00 | 34.49 | <.0001 | Hordeolum and chalazion |
| H05 | 20.28 | <.0001 | Disorders of orbit |
| H15 | 20.47 | <.0001 | Disorders of sclera |
| H60 | 20.78 | <.0001 | Otitis externa |
| H65 | 21.6 | <.0001 | Nonsuppurative otitis media |
| H66 | 21.28 | <.0001 | Suppurative and unspecified otitis media |
| H67 | 33.14 | <.0001 | Otitis media in diseases classified elsewhere |
| H68 | 22.57 | <.0001 | Eustachian salpingitis and obstruction |
| H70 | 18.48 | <.0001 | Mastoiditis and related conditions |
| H73 | 16.5 | <.0001 | Other disorders of tympanic membrane |
| H92 | 27.96 | <.0001 | Otalgia and effusion of ear |
| I87 | 35.19 | <.0001 | Other disorders of veins |
| I88 | 24.65 | <.0001 | Nonspecific lymphadenitis |
| I89 | 24.85 | <.0001 | Other noninfective disorders of lymphatic vessels and lymph nodes |
| J00 | 29.41 | <.0001 | Acute nasopharyngitis[common cold] |
| J01 | 29.6 | <.0001 | Acute sinusitis |
| J02 | 40.03 | <.0001 | Acute pharyngitis |
| J03 | 44.36 | <.0001 | Acute tonsillitis |
| J04 | 35.98 | <.0001 | Acute laryngitis and tracheitis |
| J05 | 33.88 | <.0001 | Acute obstructive laryngitis [croup] and epiglottitis |
| J06 | 37.07 | <.0001 | Acute upper respiratory infections of multiple and unspecified sites |
| J09 | 24.22 | <.0001 | Influenza due to identified zoonotic or pandemic influenza virus |
| J10 | 28.99 | <.0001 | Influenza due to identified seasonal influenza virus |
| J11 | 32.69 | <.0001 | Influenza, virus not identified |
| J12 | 21.56 | <.0001 | Viral pneumonia, NEC |
| J15 | 18.59 | <.0001 | Bacterial pneumonia, NEC |
| J16 | 19.96 | <.0001 | Pneumonia due to other infectious organisms, NEC |
| J17 | 16.48 | 0.4869 | Pneumonia in diseases classified elsewhere |
| J20 | 35.5 | <.0001 | Acute bronchitis |
| J21 | 34.71 | <.0001 | Acute bronchiolitis |
| J22 | 34.17 | <.0001 | Unspecified acute lower respiratory infection |
| J30 | 30.31 | <.0001 | Vasomotor and allergic rhinitis |
| J31 | 30.41 | <.0001 | Chronic rhinitis, nasopharyngitis and pharyngitis |
| J32 | 25.67 | <.0001 | Chronic sinusitis |
| J34 | 24.53 | <.0001 | Other disorders of nose and nasal sinuses |
| J35 | 42.9 | <.0001 | Chronic diseases of tonsils and adenoids |
| J36 | 43.56 | <.0001 | Peritonsillar abscess |
| J37 | 31.29 | <.0001 | Chronic laryngitis and laryngotracheitis |
| J38 | 21.34 | <.0001 | Diseases of vocal cords and larynx, NEC |
| J39 | 36.51 | <.0001 | Other diseases of upper respiratory tract |
| J40 | 27.39 | <.0001 | Bronchitis, not specified as acute or chronic |
| J41 | 23.93 | <.0001 | Simple and mucopurulent chronic bronchitis |
| J42 | 21.58 | <.0001 | Unspecified chronic bronchitis |
| K60 | 24.93 | <.0001 | Fissure and fistula of anal and rectal regions |
| K61 | 28.19 | <.0001 | Abscess of anal and rectal regions |
| K64 | 20.56 | <.0001 | Haemorrhoids and perianal venous thrombosis |
| K93 | 30.59 | <.0001 | Disorders of other digestive organs in diseases classified elsewhere |
| L01 | 16.79 | <.0001 | Impetigo |
| L02 | 22.89 | <.0001 | Cutaneous abscess, furuncle and carbuncle |
| L03 | 30.5 | <.0001 | Cellulitis |
| L04 | 45.94 | <.0001 | Acute lymphadenitis |
| L08 | 19.36 | <.0001 | Other local infections of skin and subcutaneous tissue |
| L60 | 25.36 | <.0001 | Nail disorders |
| L72 | 17.11 | <.0001 | Follicular cysts of skin and subcutaneous tissue |
| L84 | 16.92 | <.0001 | Corns and callosities |
| N30 | 20.08 | <.0001 | Cystitis |
| N34 | 17.99 | <.0001 | Urethritis and urethral syndrome |
| N41 | 16.88 | <.0001 | Inflammatory diseases of prostate |
| N45 | 31.22 | <.0001 | Orchitis and epididymitis |
| N49 | 18.38 | <.0001 | Inflammatory disorders of male genital organs, NEC |
| N61 | 20.16 | <.0001 | Inflammatory disorders of breast |
| N70 | 17.69 | <.0001 | Salpingitis and oophoritis |
| N71 | 17.72 | <.0001 | Inflammatory disease of uterus, except cervix |
| N73 | 19.53 | <.0001 | Other female pelvic inflammatory diseases |
| N74 | 17.69 | <.0001 | Female pelvic inflammatory disorders in diseases classified elsewhere |
| N75 | 18.99 | <.0001 | Diseases of Bartholin’s gland |
| N94 | 19.67 | <.0001 | Pain and other conditions associated with female genital organ |

**Notes:** ^a^ $P\left( NSAID prescription | ICD diagnosis \right)=\frac{Number of NSAID prescriptions issued on the day the ICD code was recorded}{Number of times the ICD codes were recorded}$

^b^ P-values were generated by chi-square test, comparing conditional prescription rate with the marginal prescription rate (16.16%).

**Abbreviations:** ICD, International Statistical Classification of Diseases, 10th Revision; NEC, Not elsewhere classified;

**Supplementary Table S4. Filtered indications by the prescription frequencies before and after the event**

| **ICD**  **code** | **Before the event** | **After the event** | **P ^a^** | **Code description** |
| --- | --- | --- | --- | --- |
| C61 | 1,699 | 1,976 | <0.001 | Malignant neoplasm of prostate |
| D10 | 1,149 | 1,456 | <0.001 | Benign neoplasm of mouth and pharynx |
| D16 | 578 | 708 | <0.001 | Benign neoplasm of bone and articular cartilage |
| D17 | 4,394 | 11,163 | <0.001 | Benign lipomatous neoplasm |
| D21 | 2,934 | 7,414 | <0.001 | Other benign neoplasms of connective and other soft tissue |
| D22 | 260 | 291 | 0.031 | Melanocytic naevi |
| D23 | 6,396 | 13,586 | <0.001 | Other benign neoplasms of skin |
| D24 | 3,721 | 3,928 | <0.001 | Benign neoplasm of breast |
| D25 | 6,084 | 6,805 | <0.001 | Leiomyoma of uterus |
| D28 | 176 | 296 | <0.001 | Benign neoplasm of other and unspecified female genital organs |
| D29 | 349 | 511 | <0.001 | Benign neoplasm of male genital organs |
| D31 | 448 | 801 | <0.001 | Benign neoplasm of eye and adnexa |
| D36 | 630 | 942 | <0.001 | Benign neoplasm of other and unspecified sites |
| E28 | 498 | 645 | <0.001 | Ovarian dysfunction |
| E29 | 453 | 576 | <0.001 | Testicular dysfunction |
| E50 | 1,455 | 1,948 | <0.001 | Vitamin A deficiency |
| E58 | 1,020 | 1,124 | <0.001 | Dietary calcium deficiency |
| G12 | 108 | 159 | <0.001 | Spinal muscular atrophy and related syndromes |
| G24 | 2,311 | 4,075 | <0.001 | Dystonia |
| G43 | 20,283 | 24,465 | <0.001 | Migraine |
| G44 | 24,811 | 32,364 | <0.001 | Other headache syndromes |
| G50 | 3,828 | 5,035 | <0.001 | Disorders of trigeminal nerve |
| G54 | 4,078 | 6,741 | <0.001 | Nerve root and plexus disorders |
| G55 | 5,683 | 6,875 | <0.001 | Nerve root and plexus compressions in diseases classified elsewhere |
| G56 | 13,442 | 26,411 | <0.001 | Mononeuropathies of upper limb |
| G57 | 10,076 | 15,696 | <0.001 | Mononeuropathies of lower limb |
| G58 | 5,423 | 7,671 | <0.001 | Other mononeuropathies |
| G59 | 719 | 893 | <0.001 | Mononeuropathy in diseases classified elsewhere |
| G62 | 5,269 | 5,783 | <0.001 | Other polyneuropathies |
| G64 | 6,573 | 9,165 | <0.001 | Other disorders of peripheral nervous system |
| G71 | 127 | 243 | <0.001 | Primary disorders of muscles |
| G90 | 3,195 | 3,984 | <0.001 | Disorders of autonomic nervous system |
| G95 | 1,438 | 1,679 | <0.001 | Other diseases of spinal cord |
| G99 | 667 | 716 | 0.031 | Other disorders of nervous system in diseases classified elsewhere |
| H00 | 15,753 | 41,008 | <0.001 | Hordeolum and chalazion |
| H01 | 21,785 | 32,132 | <0.001 | Other inflammation of eyelid |
| H02 | 5,740 | 8,158 | <0.001 | Other disorders of eyelid |
| H03 | 516 | 667 | <0.001 | Disorders of eyelid in diseases classified elsewhere |
| H04 | 58,697 | 74,531 | <0.001 | Disorders of lacrimal system |
| H05 | 1,049 | 1,595 | <0.001 | Disorders of orbit |
| H10 | 64,181 | 100,961 | <0.001 | Conjunctivitis |
| H11 | 17,734 | 27,775 | <0.001 | Other disorders of conjunctiva |
| H13 | 520 | 766 | <0.001 | Disorders of conjunctiva in diseases classified elsewhere |
| H15 | 2,974 | 5,126 | <0.001 | Disorders of sclera |
| H16 | 46,820 | 70,311 | <0.001 | Keratitis |
| H17 | 1,348 | 1,829 | <0.001 | Corneal scars and opacities |
| H18 | 4,055 | 5,849 | <0.001 | Other disorders of cornea |
| H19 | 13,565 | 18,716 | <0.001 | Disorders of sclera and cornea in diseases classified elsewhere |
| H20 | 3,962 | 8,145 | <0.001 | Iridocyclitis |
| H21 | 323 | 577 | <0.001 | Other disorders of iris and ciliary body |
| H25 | 19,750 | 31,388 | <0.001 | Senile cataract |
| H26 | 7,762 | 12,494 | <0.001 | Other cataract |
| H27 | 560 | 987 | <0.001 | Other disorders of lens |
| H28 | 135 | 222 | <0.001 | Cataract and other disorders of lens in diseases classified elsewhere |
| H30 | 1,019 | 1,103 | 0.005 | Chorioretinal inflammation |
| H31 | 1,047 | 1,242 | <0.001 | Other disorders of choroid |
| H33 | 1,231 | 1,689 | <0.001 | Retinal detachments and breaks |
| H34 | 1,637 | 1,879 | <0.001 | Retinal vascular occlusions |
| H35 | 31,227 | 41,277 | <0.001 | Other retinal disorders |
| H36 | 9,066 | 11,096 | <0.001 | Retinal disorders in diseases classified elsewhere |
| H40 | 39,466 | 53,352 | <0.001 | Glaucoma |
| H42 | 159 | 197 | 0.002 | Glaucoma in diseases classified elsewhere |
| H43 | 10,740 | 13,188 | <0.001 | Disorders of vitreous body |
| H44 | 1,536 | 1,998 | <0.001 | Disorders of globe |
| H47 | 2,032 | 2,820 | <0.001 | Other disorders of optic[2nd] nerve and visual pathways |
| H48 | 143 | 203 | <0.001 | Disorders of optic[2nd] nerve and visual pathways in diseases classified elsewhere |
| H50 | 2,470 | 3,643 | <0.001 | Other strabismus |
| H52 | 47,337 | 65,205 | <0.001 | Disorders of refraction and accommodation |
| H53 | 3,840 | 4,226 | <0.001 | Visual disturbances |
| H54 | 423 | 737 | <0.001 | Visual impairment including blindness (binocular or monocular) |
| H57 | 7,522 | 11,143 | <0.001 | Other disorders of eye and adnexa |
| H60 | 35,975 | 65,603 | <0.001 | Otitis externa |
| H61 | 11,768 | 20,710 | <0.001 | Other disorders of external ear |
| H62 | 2,707 | 4,483 | <0.001 | Disorders of external ear in diseases classified elsewhere |
| H65 | 22,150 | 28,655 | <0.001 | Nonsuppurative otitis media |
| H66 | 24,092 | 33,418 | <0.001 | Suppurative and unspecified otitis media |
| H67 | 565 | 730 | <0.001 | Otitis media in diseases classified elsewhere |
| H68 | 8,244 | 10,751 | <0.001 | Eustachian salpingitis and obstruction |
| H70 | 361 | 409 | 0.007 | Mastoiditis and related conditions |
| H72 | 1,111 | 1,732 | <0.001 | Perforation of tympanic membrane |
| H73 | 3,767 | 6,257 | <0.001 | Other disorders of tympanic membrane |
| H92 | 2,833 | 4,755 | <0.001 | Otalgia and effusion of ear |
| H93 | 15,206 | 15,733 | <0.001 | Other disorders of ear, NEC |
| I73 | 30,570 | 40,768 | <0.001 | Other peripheral vascular diseases |
| I78 | 1,035 | 1,452 | <0.001 | Diseases of capillaries |
| I80 | 2,395 | 2,676 | <0.001 | Phlebitis and thrombophlebitis |
| I83 | 4,604 | 7,441 | <0.001 | Varicose veins of lower extremities |
| I86 | 316 | 363 | 0.005 | Varicose veins of other sites |
| I87 | 16,220 | 28,575 | <0.001 | Other disorders of veins |
| I88 | 1,385 | 1,535 | <0.001 | Nonspecific lymphadenitis |
| I89 | 5,032 | 7,298 | <0.001 | Other noninfective disorders of lymphatic vessels and lymph nodes |
| I99 | 616 | 703 | <0.001 | Other and unspecified disorders of circulatory system |
| J00 | 74,622 | 111,661 | <0.001 | Acute nasopharyngitis[common cold] |
| J01 | 90,071 | 91,752 | <0.001 | Acute sinusitis |
| J02 | 74,393 | 134,126 | <0.001 | Acute pharyngitis |
| J03 | 87,505 | 166,727 | <0.001 | Acute tonsillitis |
| J04 | 100,187 | 142,160 | <0.001 | Acute laryngitis and tracheitis |
| J05 | 2,425 | 2,926 | <0.001 | Acute obstructive laryngitis [croup] and epiglottitis |
| J06 | 93,901 | 156,773 | <0.001 | Acute upper respiratory infections of multiple and unspecified sites |
| J11 | 15,817 | 17,784 | <0.001 | Influenza, virus not identified |
| J20 | 139,211 | 240,785 | <0.001 | Acute bronchitis |
| J21 | 30,983 | 33,582 | <0.001 | Acute bronchiolitis |
| J22 | 25,642 | 29,191 | <0.001 | Unspecified acute lower respiratory infection |
| J30 | 126,462 | 220,501 | <0.001 | Vasomotor and allergic rhinitis |
| J31 | 33,032 | 47,837 | <0.001 | Chronic rhinitis, nasopharyngitis and pharyngitis |
| J32 | 55,960 | 60,252 | <0.001 | Chronic sinusitis |
| J33 | 3,822 | 4,980 | <0.001 | Nasal polyp |
| J34 | 40,543 | 50,404 | <0.001 | Other disorders of nose and nasal sinuses |
| J35 | 12,548 | 20,093 | <0.001 | Chronic diseases of tonsils and adenoids |
| J36 | 20,104 | 31,258 | <0.001 | Peritonsillar abscess |
| J37 | 24,919 | 29,534 | <0.001 | Chronic laryngitis and laryngotracheitis |
| J38 | 27,068 | 28,136 | <0.001 | Diseases of vocal cords and larynx, NEC |
| J39 | 11,335 | 15,847 | <0.001 | Other diseases of upper respiratory tract |
| K35 | 1,178 | 1,509 | <0.001 | Acute appendicitis |
| K37 | 203 | 249 | <0.001 | Unspecified appendicitis |
| K40 | 1,101 | 1,187 | 0.005 | Inguinal hernia |
| K60 | 3,160 | 7,900 | <0.001 | Fissure and fistula of anal and rectal regions |
| K61 | 1,296 | 4,261 | <0.001 | Abscess of anal and rectal regions |
| K62 | 5,563 | 8,061 | <0.001 | Other diseases of anus and rectum |
| K64 | 8,559 | 18,500 | <0.001 | Haemorrhoids and perianal venous thrombosis |
| K93 | 631 | 1,058 | <0.001 | Disorders of other digestive organs in diseases classified elsewhere |
| L01 | 10,558 | 13,901 | <0.001 | Impetigo |
| L02 | 25,521 | 54,445 | <0.001 | Cutaneous abscess, furuncle and carbuncle |
| L03 | 40,199 | 94,320 | <0.001 | Cellulitis |
| L04 | 13,416 | 24,495 | <0.001 | Acute lymphadenitis |
| L05 | 568 | 865 | <0.001 | Pilonidal cyst |
| L08 | 26,157 | 46,549 | <0.001 | Other local infections of skin and subcutaneous tissue |
| L13 | 995 | 1,240 | <0.001 | Other bullous disorders |
| L55 | 145 | 207 | <0.001 | Sunburn |
| L60 | 4,772 | 12,835 | <0.001 | Nail disorders |
| L66 | 265 | 351 | <0.001 | Cicatricial alopecia [scarring hair loss] |
| L72 | 4,875 | 12,157 | <0.001 | Follicular cysts of skin and subcutaneous tissue |
| L73 | 3,396 | 4,376 | <0.001 | Other follicular disorders |
| L74 | 358 | 394 | 0.032 | Eccrine sweat disorders |
| L80 | 477 | 519 | 0.03 | Vitiligo |
| L84 | 4,357 | 10,495 | <0.001 | Corns and callosities |
| L85 | 6,167 | 6,961 | <0.001 | Other epidermal thickening |
| L86 | 100 | 123 | 0.014 | Keratoderma in diseases classified elsewhere |
| L91 | 1,514 | 1,668 | <0.001 | Hypertrophic disorders of skin |
| L92 | 323 | 549 | <0.001 | Granulomatous disorders of skin and subcutaneous tissue |
| L98 | 1,636 | 1,928 | <0.001 | Other disorders of skin and subcutaneous tissue, NEC |
| N20 | 7,476 | 11,654 | <0.001 | Calculus of kidney and ureter |
| N21 | 1,766 | 2,575 | <0.001 | Calculus of lower urinary tract |
| N23 | 894 | 1,139 | <0.001 | Unspecified renal colic |
| N30 | 29,170 | 53,547 | <0.001 | Cystitis |
| N31 | 25,678 | 32,369 | <0.001 | Neuromuscular dysfunction of bladder, NEC |
| N32 | 4,049 | 5,207 | <0.001 | Other disorders of bladder |
| N34 | 9,313 | 20,991 | <0.001 | Urethritis and urethral syndrome |
| N35 | 371 | 453 | <0.001 | Urethral stricture |
| N36 | 233 | 260 | 0.043 | Other disorders of urethra |
| N37 | 169 | 213 | <0.001 | Urethral disorders in diseases classified elsewhere |
| N39 | 19,810 | 22,429 | <0.001 | Other disorders of urinary system |
| N40 | 24,250 | 31,072 | <0.001 | Hyperplasia of prostate |
| N41 | 10,395 | 21,821 | <0.001 | Inflammatory diseases of prostate |
| N42 | 1,066 | 1,334 | <0.001 | Other disorders of prostate |
| N43 | 268 | 355 | <0.001 | Hydrocele and spermatocele |
| N45 | 1,028 | 4,030 | <0.001 | Orchitis and epididymitis |
| N48 | 1,704 | 2,512 | <0.001 | Other disorders of penis |
| N49 | 284 | 495 | <0.001 | Inflammatory disorders of male genital organs, NEC |
| N50 | 366 | 453 | <0.001 | Other disorders of male genital organs |
| N61 | 548 | 893 | <0.001 | Inflammatory disorders of breast |
| N63 | 2,784 | 3,204 | <0.001 | Unspecified lump in breast |
| N64 | 8,726 | 9,620 | <0.001 | Other disorders of breast |
| N70 | 720 | 1,084 | <0.001 | Salpingitis and oophoritis |
| N71 | 3,625 | 6,004 | <0.001 | Inflammatory disease of uterus, except cervix |
| N72 | 17,546 | 26,467 | <0.001 | Inflammatory disease of cervix uteri |
| N73 | 5,854 | 9,551 | <0.001 | Other female pelvic inflammatory diseases |
| N74 | 748 | 955 | <0.001 | Female pelvic inflammatory disorders in diseases classified elsewhere |
| N75 | 547 | 1,209 | <0.001 | Diseases of Bartholin’s gland |
| N76 | 23,086 | 34,102 | <0.001 | Other inflammation of vagina and vulva |
| N77 | 3,024 | 4,214 | <0.001 | Vulvovaginal ulceration and inflammation in diseases classified elsewhere |
| N81 | 566 | 744 | <0.001 | Female genital prolapse |
| N84 | 2,689 | 3,665 | <0.001 | Polyp of female genital tract |
| N85 | 1,116 | 1,223 | <0.001 | Other noninflammatory disorders of uterus, except cervix |
| N86 | 5,887 | 8,435 | <0.001 | Erosion and ectropion of cervix uteri |
| N87 | 2,928 | 3,052 | 0.012 | Dysplasia of cervix uteri |
| N88 | 214 | 315 | <0.001 | Other noninflammatory disorders of cervix uteri |
| N90 | 266 | 332 | <0.001 | Other noninflammatory disorders of vulva and perineum |
| N91 | 2,401 | 2,834 | <0.001 | Absent, scanty and rare menstruation |
| N92 | 1,687 | 1,985 | <0.001 | Excessive, frequent and irregular menstruation |
| N93 | 6,089 | 7,891 | <0.001 | Other abnormal uterine and vaginal bleeding |
| N94 | 938 | 1,189 | <0.001 | Pain and other conditions associated with female genital organs and menstrual cycle |
| N95 | 18,435 | 20,248 | <0.001 | Menopausal and other perimenopausal disorders |

**Notes:** ^a^ P-values were generated by Poisson test comparing prescription frequencies before and after the event

**Abbreviations:** ICD, International Statistical Classification of Diseases, 10th Revision; NEC, Not elsewhere classified;**Supplementary Table S5. Lists of NSAIDs in our study**

| **ATC code** | **Drug name** |
| --- | --- |
| M01AB16 | Aceclofenac |
| M01AB11 | Acemetacin |
| M01AE16 | Alminoprofen |
| M01AX07 | Benzydamine |
| M01AH01 | Celecoxib |
| M01AE14 | Dexibuprofen |
| M02AA27 | Dexketoprofen |
| M01AE17 | Dexketoprofen |
| M01AB05 | Diclofenac |
| N02BA11 | Diflunisal |
| N02BA07 | Ethenzamide |
| M01AB08 | Etodolac |
| M01AB09 | Etodolac |
| M01AB10 | Etodolac |
| M01AB11 | Etodolac |
| M01AB12 | Etodolac |
| M01AB13 | Etodolac |
| M01AE04 | Fenoprofen |
| M01AB10 | Fentiazac |
| M01AG03 | Flufenamic Acid |
| M01AE09 | Flurbiprofen |
| M01AE01 | Ibuprofen |
| M01AE13 | Ibuproxam |
| N02BA16 | Imidazole Salicylate |
| M01AB01 | Indomethacin |
| M02AA23 | Indomethacin |
| M01AE03 | Ketoprofen |
| M02AA10 | Ketoprofen |
| M01AE53 | Ketoprofen |
| M01AB15 | Ketorolac |
| M01AB09 | Lonazolac |
| M01AC05 | Lornoxicam |
| M01AE | Loxoprofen Sodium |
| M01AC02 | Meloxicam |
| M01AX22 | Morniflumate |
| M01AX01 | Nabumetone |
| M01AE02 | Naproxen |
| M01AX17 | Nimesulide |
| M01AE12 | Oxaprozin |
| M01AC01 | Piroxicam |
| M01AH07 | Polmacoxib |
| M01AB14 | Proglumetacin |
| M01AB02 | Sulindac |
| M01AC06 | Tenoxicam |
| M01AE11 | Tiaprofenic Acid |
| M01AG02 | Tolfenamic Acid |
| M02AA21 | Tolmetin |
| M01AB03 | Tolmetin |
| M01AE | Zaltoprofen |

**Abbreviations:** ATC, Anatomical therapeutic chemical

**Supplementary Table S6. Considered concomitant drugs in sensitivity analyses**

| ICD code | Confounding drugs |
| --- | --- |
| D50 | Anti-platelet and Anti-coagulation agents, aspirin |
| D62 | Anti-platelet and Anti-coagulation agents, aspirin |
| D64 | Anti-platelet and Anti-coagulation agents, aspirin |
| E87 | RAAS inhibitors, diuretics |
| I10 |  |
| I50 | RAAS inhibitors |
| I63 | Anti-platelet and Anti-coagulation agents, aspirin, statin |
| N17 | RAAS inhibitors |

**Abbreviations**: ICD, International Statistical Classification of Diseases, 10th Revision; RAAS, Renin-angiotensin-aldosterone system

**
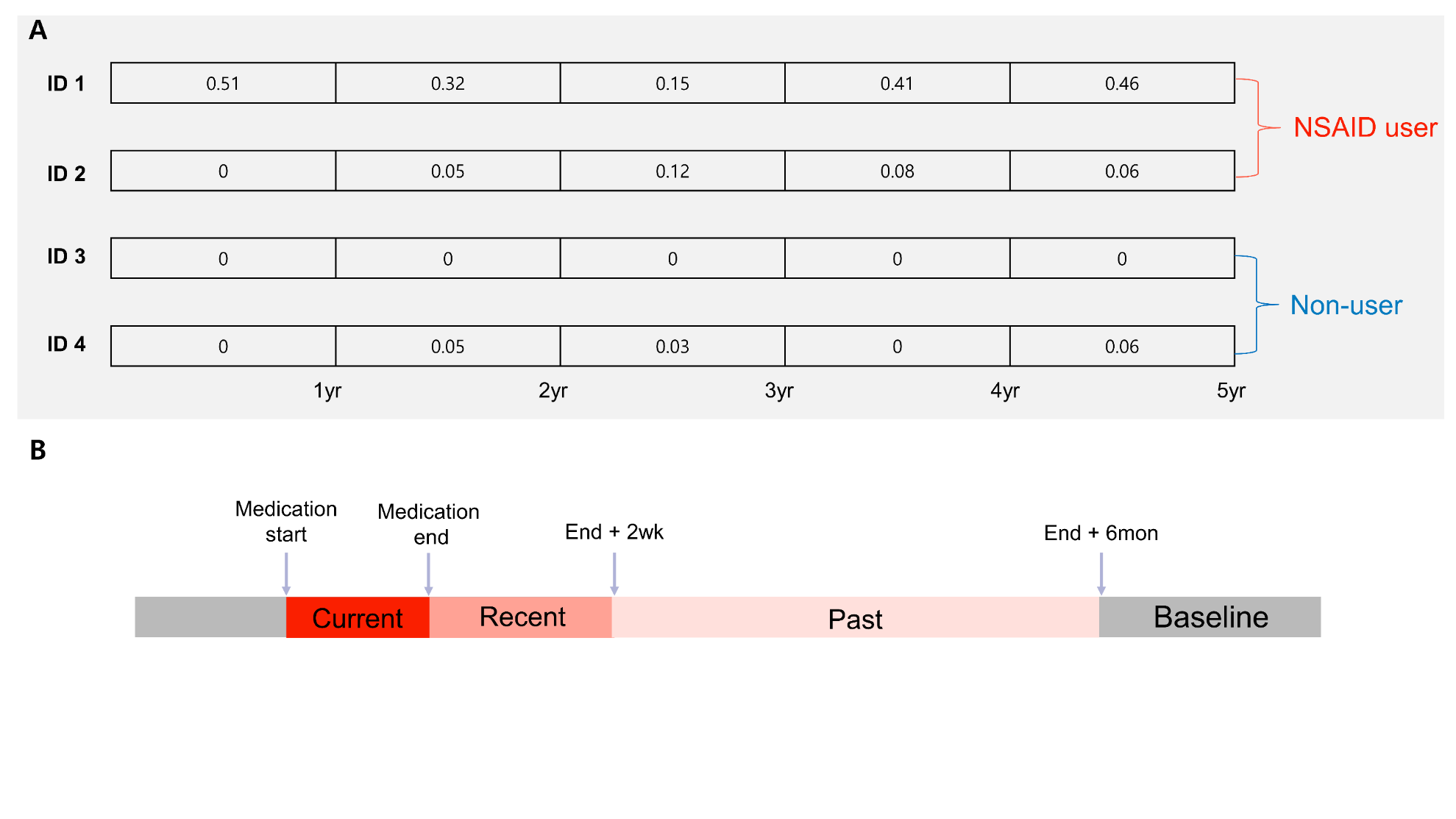
**

**Supplementary Figure S1. Diagram illustrating the concept of exposure**

In the cohort analysis, NSAID users and non-users were classified by annual MPR. Patients with an annual MPR of 0.1 or higher at least once were assigned to the medication group, while others were to the control group, and (A) represents the example.

(B) illustrates the definition of the risk periods in the case-only study, with the medication period, the subsequent 2 weeks, and the following 6 months defined as current, recent, and past periods, respectively.

**Abbreviations:** NSAID, Non-Steroidal Anti-Inflammatory Drugs; MPR, Medication possession ratio;


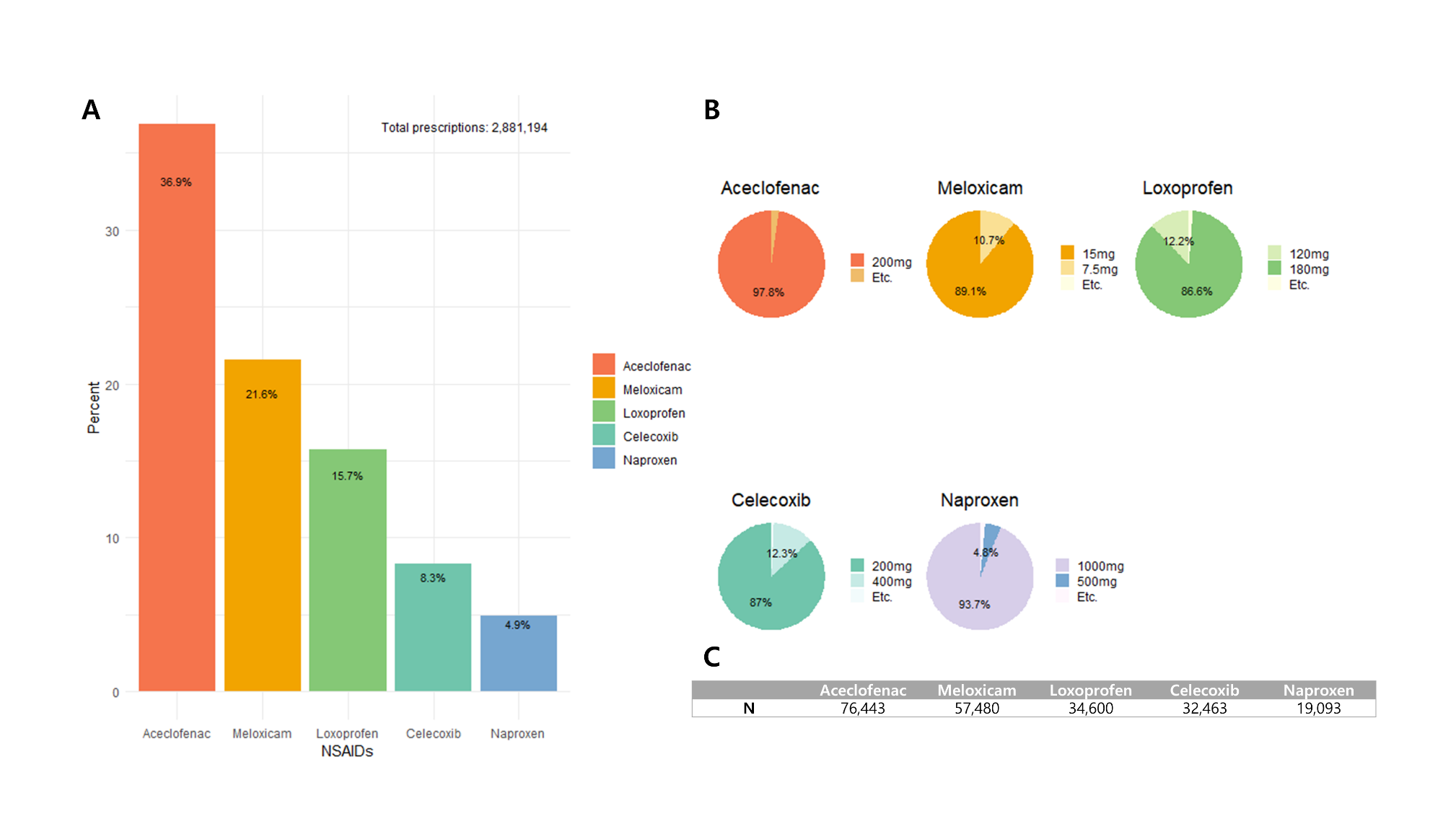


**Supplementary Figure S2.** **Frequency and Daily Dose of Commonly Prescribed NSAIDs**

(A) NSAID prescription distribution

(B) Daily doses

(C) Sample sizes for each subtype

**Abbreviations:** NSAID, Non-Steroidal Anti-Inflammatory Drugs;


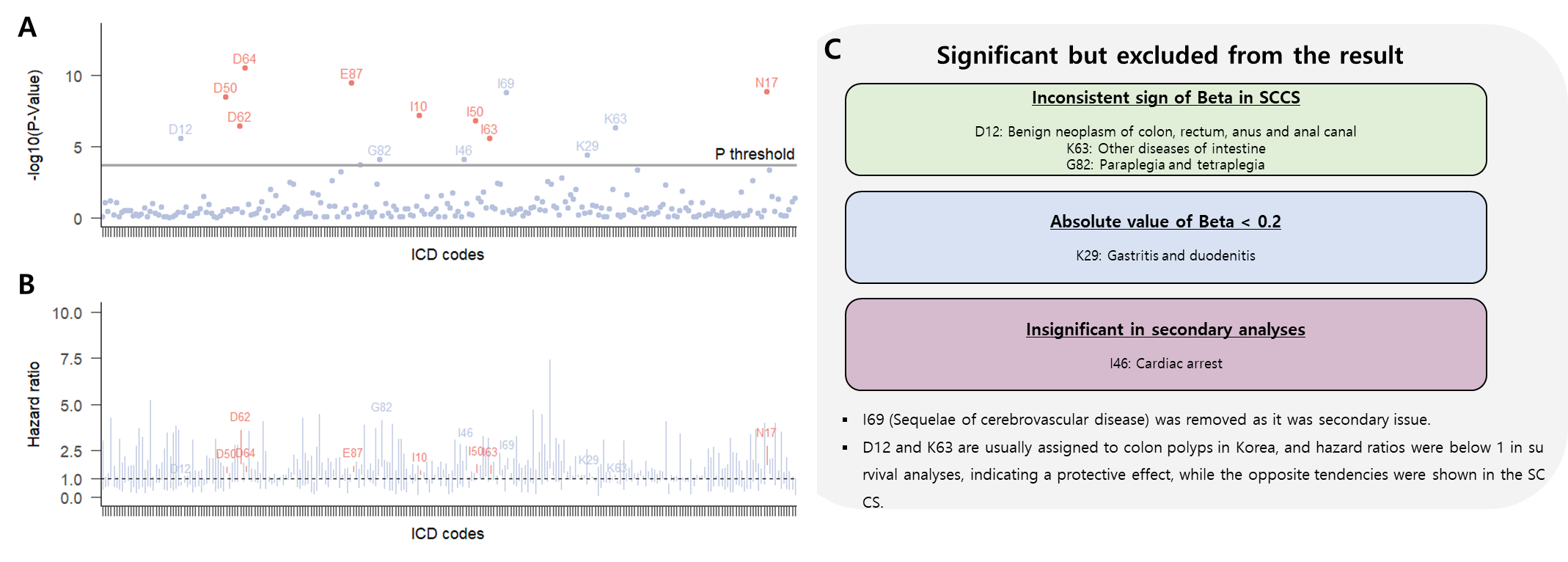
 **Supplementary Figure S3.** **Results for all ICD codes in cohort study**

Figures (A) and (B) show raw P-values and hazard ratios for each ICD code. In Figure (A), with a P threshold of 0.0002, only the red points above the P threshold line were chosen as the final outcome of our study. Blue points above the line were excluded for several reasons, which are explained in Figure (C).

**Abbreviations**: NSAID, Non-Steroidal Anti-Inflammatory Drugs; ICD, International Statistical Classification of Diseases, 10th Revision; SCCS, Self-Controlled case series study;


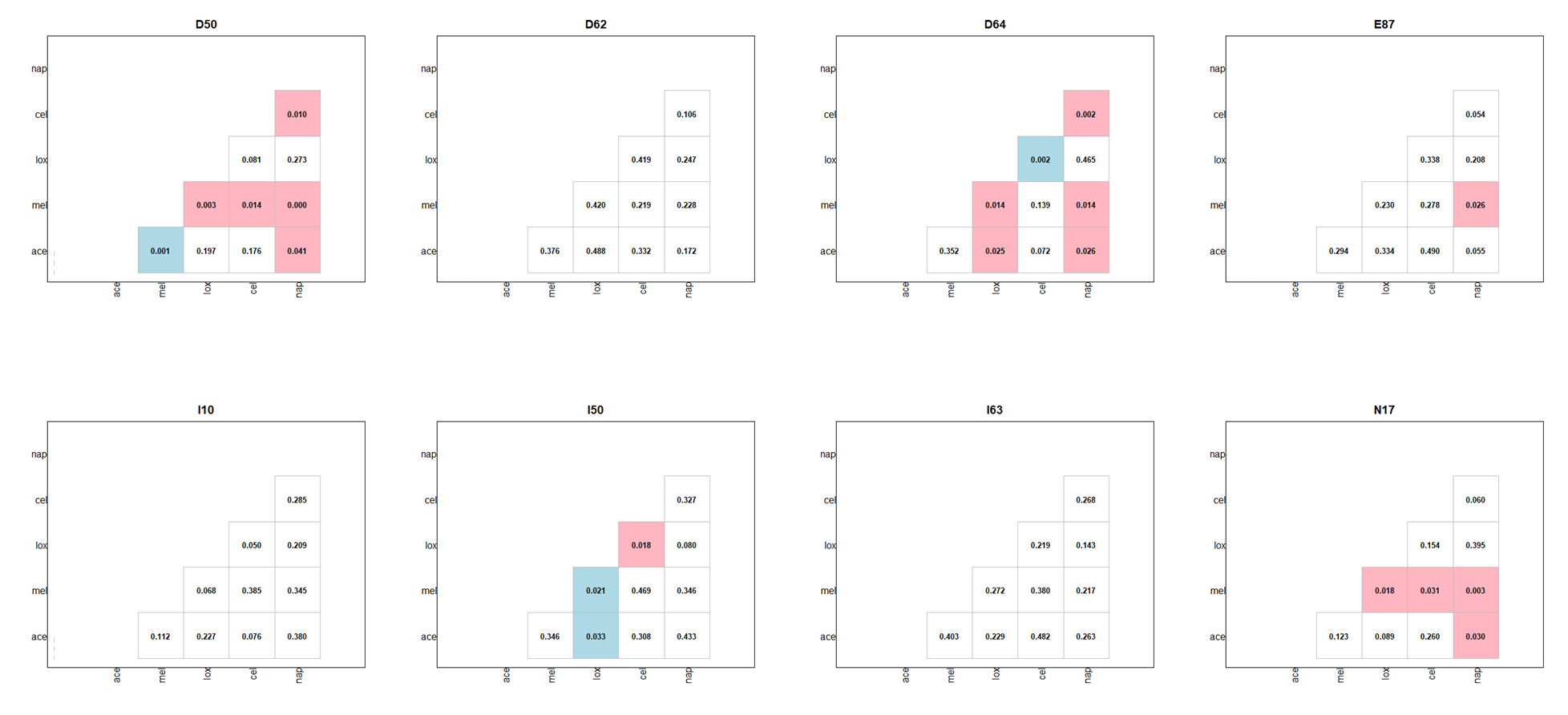


**Supplementary Figure S4.** **Pairwise comparison of hazard ratios for each ICD code**

Values in the figure represent P-values from pairwise comparisons. If the HR of the drug is significantly higher based on the x-axis, it is colored red; if significantly lower, it is colored blue.

**Abbreviations**: Ace, Aceclofenac; Mel, Meloxicam, Lox, Loxoprofen; Cel, Celecoxib; Nap, Napoxen;


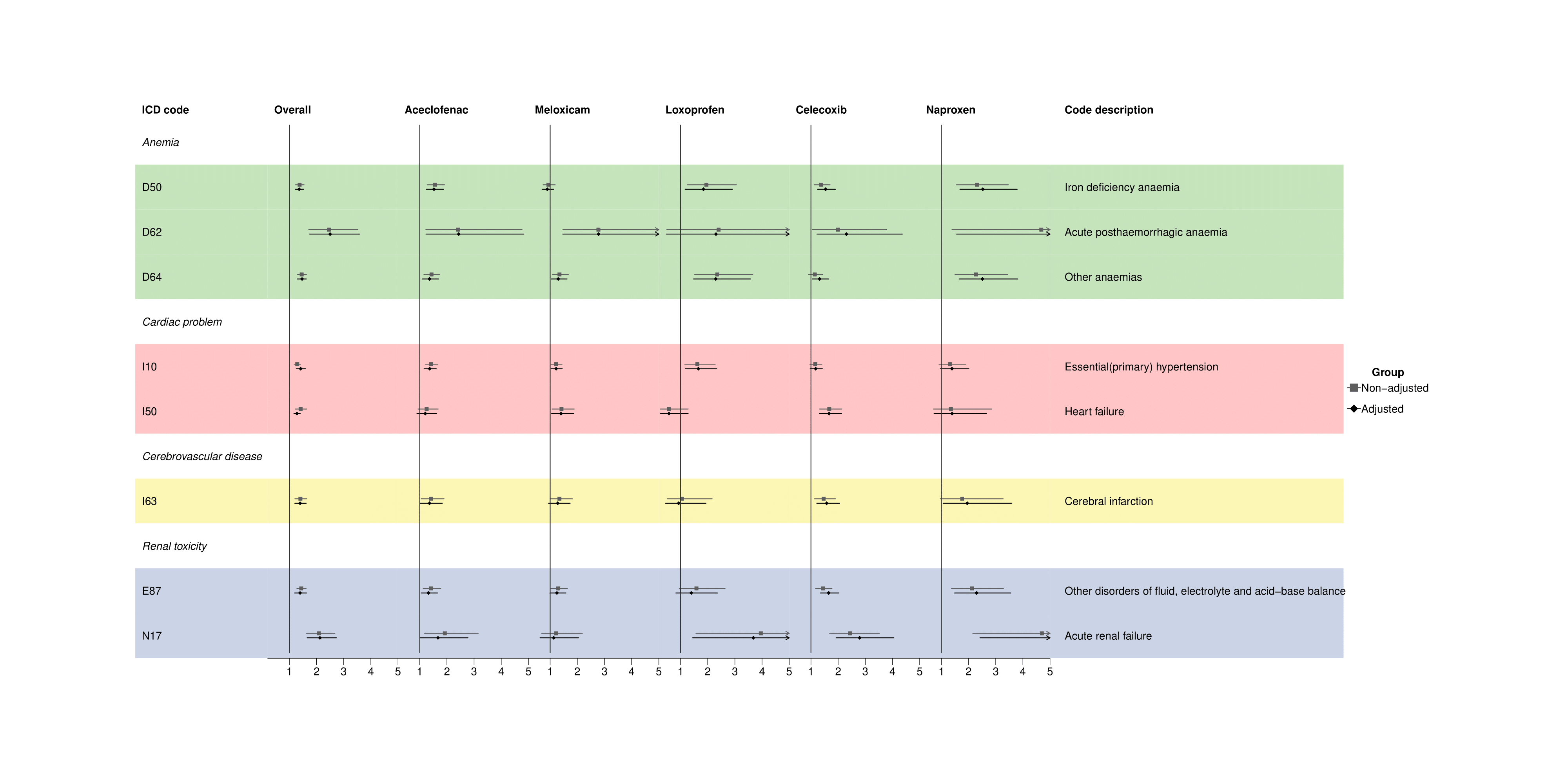


**Supplementary Figure S5.** **Overview of hazard ratios in subtype analyses**

Hazard ratios for MPR are shown. ‘Adjusted’ means results obtained after incorporating additional adjustments for confounders (smoking and alcohol consumption) to each. On the other hand, ‘Non-adjusted’ indicates that conducted only common adjustments (age, sex, body mass index, income, residence, and the Charlson comorbidity index).

**Abbreviations**: ICD, International Statistical Classification of Diseases, 10th Revision; MPR, medication possession ratio;
